# Sites of synchronous distant metastases and prognosis in prostate cancer patients with bone metastases at initial diagnosis: a population-based study of 16,643 patients

**DOI:** 10.1186/s40169-019-0247-4

**Published:** 2019-11-29

**Authors:** Feng Zhao, Jili Wang, Meiqin Chen, Danni Chen, Sunyi Ye, Xinke Li, Xin Chen, Guoping Ren, Senxiang Yan

**Affiliations:** 1 Department of Radiation Oncology, The First Affiliated Hospital, College of Medicine, Zhejiang University, Hangzhou, 310003 Zhejiang People’s Republic of China; 2 Graduate School, College of Medicine, Zhejiang University, Hangzhou, 310003 Zhejiang People’s Republic of China; 3 Department of Urology, The First Affiliated Hospital, College of Medicine, Zhejiang University, Hangzhou, 310003 Zhejiang People’s Republic of China; 4 Institute of Pharmaceutical Biotechnology and the First Affiliated Hospital, College of Medicine, Zhejiang University, Hangzhou, 310058 Zhejiang People’s Republic of China; 5 Department of Pathology, The First Affiliated Hospital, College of Medicine, Zhejiang University, Hangzhou, 310003 Zhejiang People’s Republic of China

**Keywords:** Prostate cancer, Metastasis, Surgery, Radiotherapy (RT), Chemotherapy, Overall survival (OS), Prognosis

## Abstract

**Background:**

Bone is a preferential site for prostate cancer (PCa) metastasis. However, sites of synchronous distant metastases in PCa patients with bone metastases at initial diagnosis and their impacts on prognosis are still unclear, limiting our ability to better stratify and treat the patients. In this study, we examined the sites of synchronous extra-skeletal metastases in de novo PCa patients with bone metastases and their associated prognoses.

**Methods:**

In total, 16,643 de novo PCa patients with bone metastases from the SEER database were included. After stratification of metastatic sites (bone, lung, liver, and brain) and treatment modalities, overall survival (OS) and independent predictors of OS, were analyzed.

**Results:**

Lung was the most frequent site of synchronous metastases, followed by liver, while brain metastases were relatively uncommon. Patients with bone-only metastases showed the longest mean survival time (35.87 months, p < 0.001), followed by patients with bone and lung metastases (30.74 months, p < 0.001). Patients with bone and liver metastases had the shortest mean survival time (17.39 months, p < 0.001). Age > 70 years, unmarried status, high tumor grade, prostate-specific antigen (PSA) > 50 ng/ml, and Gleason score ≥ 8 were associated with poor OS (all p < 0.01). Asian or Pacific Islander ethnic background was associated with a favorable OS (all p < 0.01). Chemotherapy improved OS in patients without brain metastases (all p < 0.05). For patients with bone-only metastases, radical prostatectomy (RP) (HR, 0.339; 95% CI 0.231–0.495; p < 0.001), brachytherapy (BT) (HR, 0.567; 95% CI 0.388–0.829; p = 0.003), and chemotherapy (HR, 0.850; 95% CI 0.781–0.924; p < 0.001) were associated with prolonged OS.

**Conclusions:**

Age, race, tumor grade, PSA, Gleason score, sites of synchronous extra-skeletal metastases, as well as treatment modalities affected OS in newly diagnosed PCa patients with bone metastases. Synchronous liver metastases were associated with poor OS. Chemotherapy improved OS in patients without brain metastases. RP and BT improved OS in patients with bone-only metastases. Further investigation is warranted to validate these findings.

## Background

Prostate cancer (PCa) is the second most commonly diagnosed cancer in men, with an estimated 1.1 million diagnoses worldwide in 2012, accounting for 15% of all diagnosed cancers [1, 2]. Approximately 1.7–11.9% of patients have bone metastases at initial diagnosis, and the incidence varies greatly in different countries or regions, which may attribute to the adoption of different prostate-specific antigen (PSA) screening policies [3–5]. The bone is the most common distant metastatic site of PCa and many existing literatures have focused on the topic and reported the prognosis of this subgroup of PCa patients [5–7]. For example, Norgaard et al. [4] reported that the 1-year and 5-year survival rates were approximately 47% and 3%, respectively, for PCa patients with bone metastases at initial diagnosis. Apart from PCa, breast and kidney cancers also have a propensity of metastasizing to the bones; and patients with disease that remains confined to the skeleton have a better prognosis than do those with synchronous multiple sites of involvement [8, 9]. However, the sites of synchronous extra-skeletal metastases, e.g., the lung, brain, and liver, and the associated prognostic outcomes in de novo PCa patients with bone metastases have not been thoroughly investigated.

With regard to treatment, androgen deprivation therapy (ADT) has been the backbone of treatment in de novo PCa patients with bone metastases. Recently, ADT plus chemotherapy (docetaxel) or abiraterone acetate are also recommended as first-line treatment options for metastatic PCa patients [10, 11]. Most recently, the European Association of Urology (EAU) 2019 and National Comprehensive Cancer Network (NCCN) 2019 V2 clinical practice guidelines recommended that ADT combined with prostate radiotherapy (RT) be offered to patients who first present with a low volume metastasis (M1) according to the Chemohormonal Therapy Versus Androgen Ablation Randomized Trial for Extensive Disease in Prostate Cancer (CHAARTED) criteria and STAMPEDE phase 3 randomized trial, which randomized 2061 patients based on the mean duration to standard systemic therapy with or without radiotherapy to the primary tumor [10, 11]. However, until now, local definitive surgery has not been a part of the treatment recommendation for newly diagnosed PCa with bone metastases, although some studies have suggested that local resection of the primary cancer might be beneficial to prolonging the survival rate of patients [12–14].

Therefore, the primary objective of this study was to investigate the sites of synchronous distant metastases of other organs (lung, brain, and liver) and the associated prognoses in de novo PCa patients with bone metastases, using data from the Surveillance, Epidemiology, and End Results (SEER) Program of the National Cancer Institute. The secondary aim was to investigate the survival benefits of surgery, RT, and chemotherapy in patients through the stratification of metastatic sites and treatment modalities.

## Patients and methods

### Study population

The SEER database of the National Cancer Institute was used for this analysis. The SEER program, which consists of 18 population-based cancer registries (released in November 2018) and covers approximately 26% of the sample of the United States population, is representative of the United States in terms of demographics, tumor, diagnostics and treatment characteristics.

**Fig. 1:**
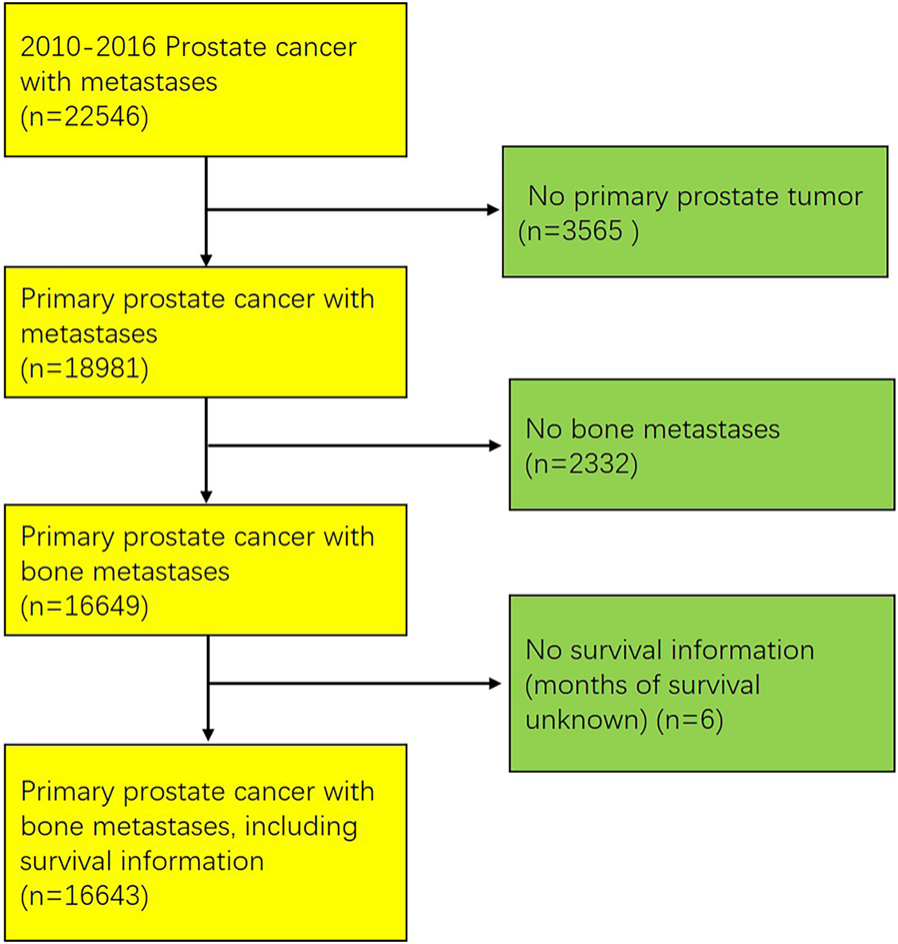
This flow chart describes the steps taken to identify cases in the SEER database of de novo primary prostate cancer patients with bone metastases

PCa patients diagnosed between 2010 and 2016 were selected from the database. The eligibility criteria included the following: (1) distant metastases (M1 for patients diagnosed between 2010 and 2015 (the 7th AJCC M stage), cM1 or pM1 for patients diagnosed in 2016 (derived-SEER combined M stage)); (2) tumor sequence number labeled “one primary only”; (3) bone metastases; and (4) survival information available. Finally, a total of 16,643 eligible patients were included in this study (Fig. 1).

### Variable definitions

Patient demographic variables, including age at diagnosis, marital status, and race, were extracted from the database. There are 6 marital statuses in the SEER database: married, single, widowed, divorced, separated and unmarried or domestic partner. Only “married” patients were included in the married cohort. Others such as “single”, “divorced”, “widowed”, “separated” or “unmarried or domestic partner” were categorized as “unmarried”. Tumor factors, including tumor grade, serum PSA, Gleason Score, and metastatic site (bone, brain, liver, and lung), as well as treatment modalities, such as surgery, radiotherapy, and chemotherapy, were obtained from the database. Furthermore, surgery was also subcategorized as endoscopic therapy (surgery site codes 10–30) and radical prostatectomy (RP) (surgery site codes 50 or 70), while radiotherapy was subclassified as brachytherapy (BT) (radiation-specific codes 2, 3, or 4) and external-beam radiation (EBRT) (radiation-specific code 1). Additionally, the overall survival (OS) status and information on the months (M) of survival were collected for survival analysis.

### Statistical analyses

Categorical variables are reported as counts (percentages). PCa patients with bone metastases were classified according to the sites of metastases (bone-only, bone and lung, bone and liver, bone and brain, and bone metastases and ≥ 2 other sites). Kaplan–Meier analysis and log-rank tests were used to estimate the survival times of patients. Landmark analysis was performed when survival curves cross each other. Univariable and multivariable Cox proportional hazards models were used to analyze the associations of patient characteristics, the sites of metastases and treatment modalities with patient survival. Hazard ratios (HRs) and 95% confidence intervals (CIs) were estimated. Statistical analyses were performed with SPSS version 25.0 (SPSS Inc., Chicago, IL, USA) and EmpowerStats statistical software (http://www.empowerstats.com/). A p value < 0.05 was considered statistically significant. Forest plots were generated using the forestplot package in R software (version 3.5.3).

## Results

The demographic and clinicopathological characteristics of the patients are summarized in Table 1. The median age was 71 years old. Most patients (74.3%) were White, and more than half of the patients (54.0%) were married. The majority of patients (90.56%) with available tumor grade information had poorly differentiated diseases. For the patients with available PSA information, most (69.44%) had PSA > 50 ng/ml. For the patients with available Gleason information, the majority (83.08%) had Gleason scores of 8–10. A minority of patients with bone metastasis had concomitant lung metastases (5.9%), liver metastasis (2.5%), brain metastasis (0.8%), and 2 or more other sites (brain, liver, and lung) of metastases (1.3%). Patient with bone metastases received treatment modalities including surgery (9.6%), RT (22.6%), and chemotherapy (13.3%).

**Table 1:** Patient demographics and clinical characteristics of de novo prostate cancer patients with bone metastases (N = 16,643)

Characteristics	Level	Number (%)
Age at diagnosis	Mean ± SD	71.41 ± 11.318
	Median (range)	71 (34–105)
	≤ 70	8079 (48.5%)
	> 70	8564 (51.5%)
Race	White	12,367 (74.3%)
	Black	303 (18.2%)
	Asian or Pacific Islander	989 (5.9%)
	Others/unknown	257 (1.5%)
Marital status	Married	8987 (54.0%)
	Unmarried	6542 (39.3%)
	Unknown	1114 (6.7%)
Tumor grade	Well differentiated	96 (0.6%)
	Moderately differentiated	861 (5.2%)
	Poorly differentiated	9097 (54.6%)
	Undifferentiated	87 (0.5%)
	Unknown	6502 (39.1%)
PSA	≤ 10 ng/ml	1271 (7.6%)
	10–20 ng/ml	1240 (7.4%)
	20–50 ng/ml	2037 (12.2%)
	> 50 ng/ml	10,336 (62.1%)
	Unknown	1759 (10.6%)
Gleason	≤ 6	219 (1.3%)
	3 + 4	536 (3.2%)
	4 + 3	885 (5.3%)
	8–10	8052 (48.4%)
	Unknown	6951 (41.8%)
Metastatic sites	Bone-only	14,872 (89.4%)
	Bone and lung	976 (5.9%)
	Bone and liver	414 (2.5%)
	Bone and brain	126 (0.8%)
	Bone and ≥ 2 other sites	255 (1.3%)
Surgery	Yes	1593 (9.6%)
	No/unknown	15,085 (90.4%)
Radiotherapy	Yes	3765 (22.6%)
	No/unknown	12,878 (77.4%)
Chemotherapy	Yes	2216 (13.3%)
	No/unknown	14,427 (86.7%)

*PSA* prostate specific antigen

**Fig. 2:**
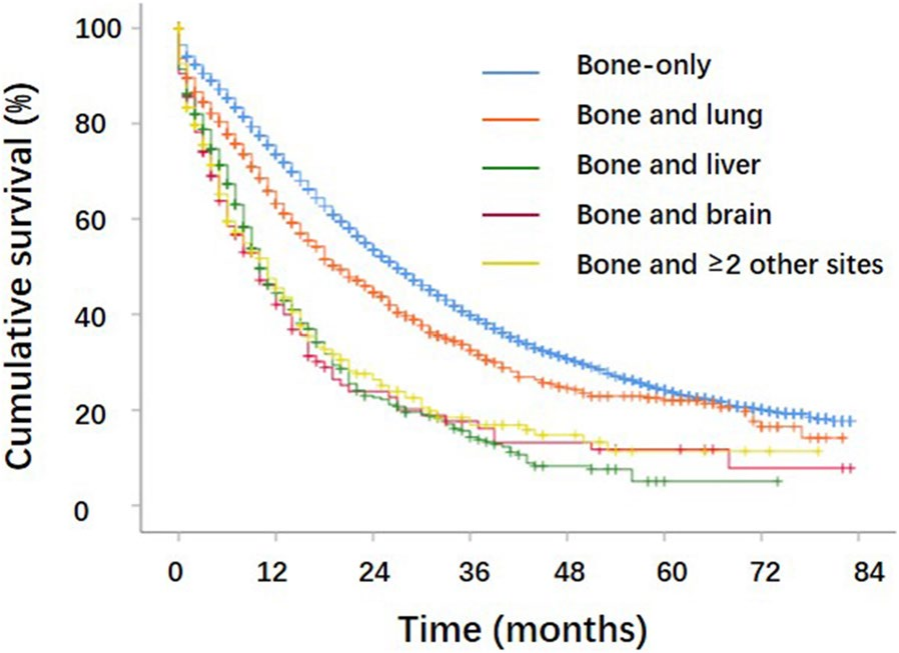
Kaplan-Meier survival curves and log-rank tests showing that overall survival (OS) differences between prostate cancer patients with bone-only metastases, bone and lung metastases, bone and liver metastases, bone and brain metastases, and bone and ≥ 2 other sites metastases (i.e., bone-only vs bone and lung, bone-only vs bone and liver, bone-only vs bone and brain, and bone-only vs bone and ≥ 2 other sites, all p < 0.001; bone and lung vs bone and liver, bone and lung vs bone and brain, bone and lung vs bone and ≥ 2 other sites, all p < 0.001; bone and liver, bone and brain, and bone and ≥ 2 other sites, compared with each other, all p > 0.05)

**Fig. 3:**
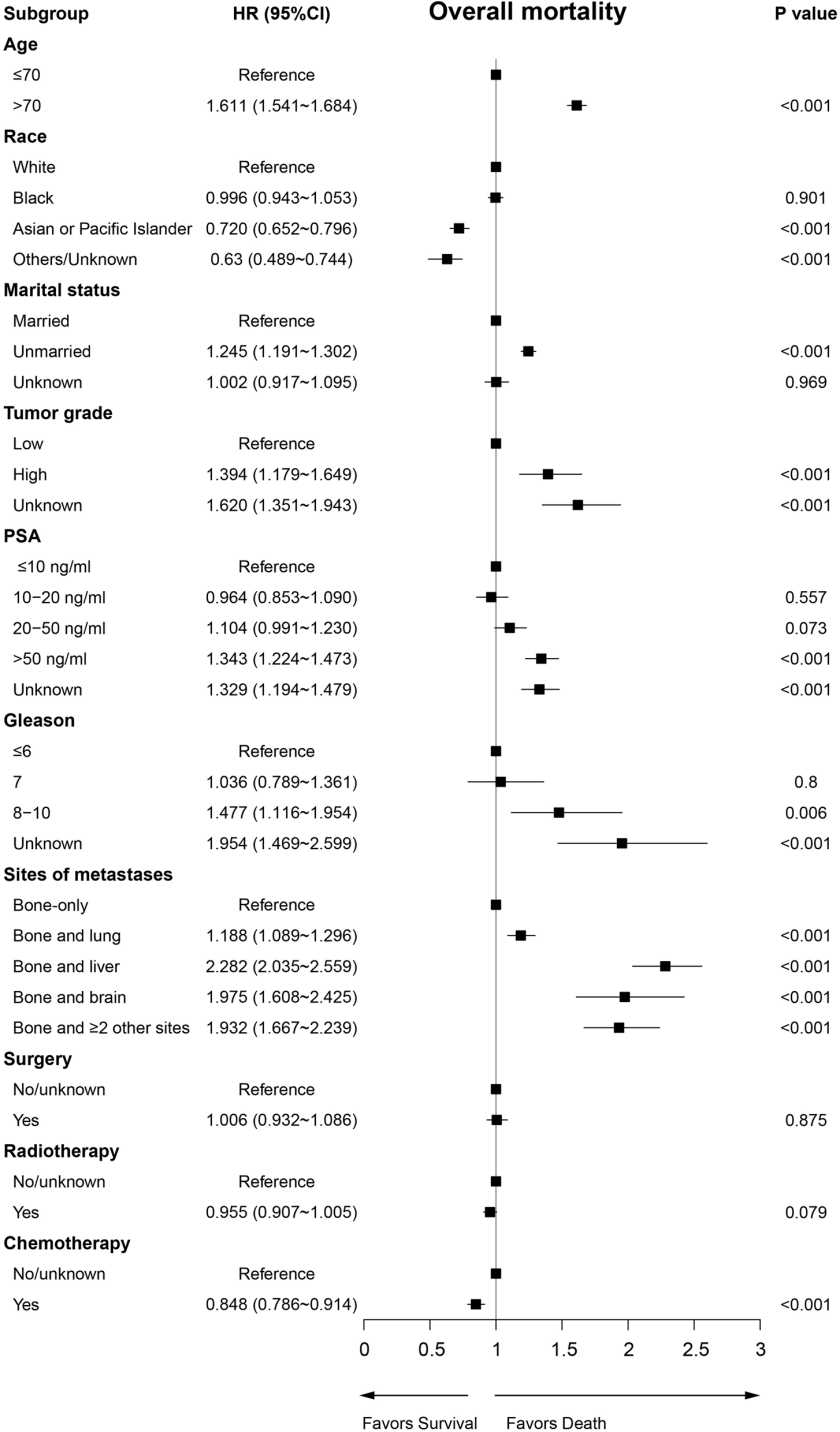
Multivariable Cox proportional hazards models were used to analyze associations of patients’ characteristics, sites of metastases and treatment modalities against survival in de novo PCa patients with bone metastases. Hazard ratios (HRs) and 95% confidence intervals (CIs) were estimated and summarized with forest plots

As shown in Table 2 and Fig. 2, patients with bone-only metastases showed the longest mean survival time (35.87 months, all p < 0.001), followed by bone and lung metastases (30.74 months, all p < 0.001). Patients with bone and liver metastases had the shortest mean survival time (17.39 months, p < 0.001). The risks of mortality for PCa patients with different types of metastases are illustrated in Fig. 3. Specifically, as shown in Fig. 3, older age (age > 70 years), an unmarried status, high tumor grade, PSA > 50 ng/ml, and Gleason score ≥ 8 were associated with a worse OS (all p < 0.01), whereas an Asian or Pacific Islander ethnic background was associated with a better OS (p < 0.001). As to treatment modalities, only chemotherapy reduced the risk of death (HR, 0.848; 95% CI 0.786–0.914; p < 0.001). Both surgery (HR, 1.006; 95% CI 0.932–1.086; p = 0.875) and RT (HR, 0.955; 95% CI 0.907–1.005; p = 0.079) had little effect on survival.

**Table 2:** Accumulated overall survival (OS) rates (median, mean, 1-, 2-, and 5-year) of de novo prostate cancer patients with bone metastases

Metastatic sites	Median survival (months)	Mean survival (months)	1-year (%)	2-year (%)	5-year (%)
Bone-only	27	35.87	73.6	53.4	24.2
Bone and lung	20	30.74	63.2	44.6	22.1
Bone and liver	10	17.39	44.5	22.6	5.1
Bone and brain	10	19.11	42.0	23.9	11.8
Bone and ≥ 2 other sites	11	20.23	45.5	26.4	11.4

**Fig. 4:**
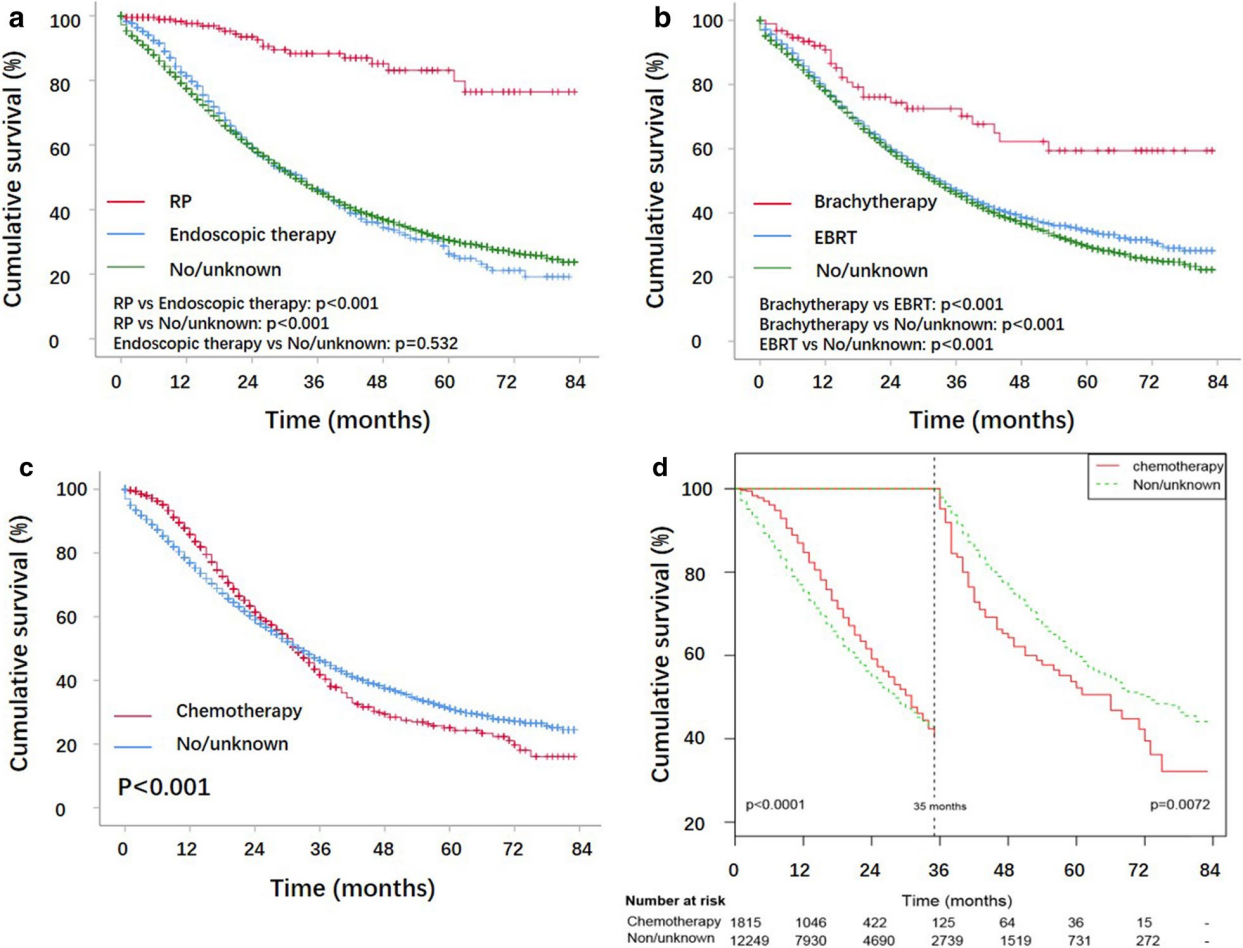
Kaplan-Meier survival curves and log-rank tests showing OS in prostate cancer patients with bone-only metastases. **a** OS differences between patients who underwent RP, endoscopic therapy and non/unknown surgery. **b** OS differences between patients who underwent brachytherapy, EBRT and non/unknown RT. **c** OS differences between patients who underwent chemotherapy and non/unknown chemotherapy. **d** Landmark analysis discriminating between patients who underwent chemotherapy and non/unknown chemotherapy

The HRs and 95% CIs of the overall mortality for patients are summarized in Table 3, according to the stratification of metastatic sites and treatment modalities. Chemotherapy improved OS in patients with bone-only metastases (HR, 0.850; 95% CI 0.781–0.924; p < 0.001), bone and lung metastases (HR, 0.737; 95% CI 0.552–0.984; p = 0.039), and bone and liver metastases (HR, 0.651; 95% CI 0.491–0.864; p = 0.003). To better understand the contribution of surgery and RT to OS, we subclassified the surgical treatment as radical surgery (RP), endoscopic therapy and no/unknown surgery and subdivided RT into BT, EBRT and no/unknown RT for further analysis. For the patients with bone-only metastases, RP (HR, 0.339; 95% CI 0.231–0.495; p < 0.001) and BT (HR, 0.567; 95% CI 0.388–0.829; p = 0.003) significantly prolonged OS, while endoscopic therapy was associated with a worse OS. EBRT had no effect on OS. The Kaplan–Meier OS curves for the patients with bone-only metastases are shown in Fig. 4, which revealed that patients who received RP had a significantly longer OS than those underwent endoscopic therapy and no/unknown surgery. Patients who received BT had a significantly longer OS than those underwent EBRT and no/unknown RT. Interestingly, stratified by chemotherapy, Fig. 4c showed the Kaplan–Meier survival curves cross each other at point of 35 months. Thus, landmark analysis (Fig. 4d) was performed and showed that, in patients with bone-only metastases, those who received chemotherapy had a significantly longer OS than those who did not before the landmark point of 35 months. However, significantly shorter OS after 35 months was otherwise observed in those patients receiving chemotherapy.

**Table 3:** Univariate and multivariate Cox regression for all-cause mortality based on treatment modalities and metastatic sites

Groups	Variables	Level	n	Univariate		Multivariate	
				HR (95% CI)	p value	HR (95% CI)	p value
Bone-only (n = 14,872)	Surgery	No/unknown	13,475	1		1	
		Endoscopic therapy	1191	0.975 (0.898–1.058)	0.539	1.106 (1.016–1.204)	0.020
		RP	206	0.187 (0.128–0.274)	< 0.001	0.339 (0.231–0.495)	< 0.001
	RT	No/unknown	11,584	1		1	
		EBRT	3194	0.898 (0.850–0.949)	< 0.001	0.954 (0.902–1.009)	0.097
		Brachytherapy	94	0.396 (0.272–0.578)	< 0.001	0.567 (0.388–0.829)	0.003
	Chemo	No/unknown	13,015	1		1	
		Yes	1857	0.761 (0.700–0.827)	< 0.001	0.850 (0.781–0.924)	< 0.001
Bone and lung (n = 976)	Surgery	No/unknown	899	1			
		Endoscopic therapy	72	0.844 (0.609–1.169)	0.308		
		RP	5	–	–		
	RT	No/unknown	758	1			
		EBRT	216	0.915 (0.744–1.125)	0.400		
		Brachytherapy	2	–	–		
	Chemo	No/unknown	808	1		1	
		Yes	168	0.643 (0.483–0.855)	0.002	0.737 (0.552–0.984)	0.039
Bone and liver (n = 414)	Surgery	No/unknown	355	1			
		Endoscopic therapy	57	0.970 (0.707–1.332)	0.852		
		RP	2	–	–		
	RT	No/unknown	333	1			
		EBRT	80	0.898 (0.673–1.198)	0.464		
		Brachytherapy	1	–	–		
	Chemo	No/unknown	306	1		1	
		Yes	108	0.629 (0.477–0.830)	0.001	0.651 (0.491–0.864)	0.003
Bone and brain (n = 126)	Surgery	No/unknown	122	1			
		Endoscopic therapy	2	–	–		
		RP	2	–	–		
	RT	No/unknown	78	1			
		EBRT	46	1.015 (0.668–1.541)	0.944		
		Brachytherapy	2	–	–		
	Chemo	No/unknown	107	1			
		Yes	19	0.577 (0.314–1.062)	0.077		
Bone and ≥ 2 other sites (n = 255)	Surgery	No/unknown	234	1			
		Endoscopic therapy	21	0.872 (0.474–1.606)	0.661		
	RT	No/unknown	184	1			
		EBRT	71	1.007 (0.730–1.390)	0.965		
	Chemo	No/unknown	191	1			
		Yes	64	1.009 (0.709–1.43600)	0.959		

Multivariate analysis was adjusted for age, marriage, race, PSA, and Gleason score

## Discussion

### Younger age and married status were associated with prolonged OS

Previous studies based on the SEER database have reported that the median age of localized and locally advanced PCa patients are 61 and 65 years, respectively [15, 16]. Our study found that PCa patients with bone metastases were significantly older than those with localized and locally advanced PCa patients, with a median age of 71 years. Meanwhile, older age (age > 70 years) was associated with a poor prognosis, consistent with previous reports [12, 17, 18].

Studies based on the SEER database have reported that the married proportion of localized and locally advanced PCa patients are 77% and 71%, respectively [15, 16]. In our study, the proportion of married PCa patients with bone metastases was lower than that of localized and locally advanced PCa patients (54% vs 77% and 71%). Conceivably, this is because PCa patients with bone metastases were older and more likely to be widowed than younger patients. Meanwhile, an unmarried status was also associated with a worse survival, possibly due to married patients more easily to receive social and financial support from their families and to choose a proactive treatment. As reported in previous studies, marital status is a prognostic factor for the better survival in PCa patients [19, 20].

In addition, our study indicated that high pathological grades, high PSA levels (> 50 ng/ml), and high Gleason scores (Gleason score ≥ 8) of the tumor were associated with a worse OS. These findings are in line with those of former studies [12, 17, 21].

### Asian or Pacific Islander ethnic background was associated with favorable OS

Several studies have reported that for localized and locally advanced PCa patients, Black men consistently have a higher mortality rate than do men of other ethnicities. One possible reason for this finding is that Black men are less likely to be treated with a curative intent than are White men [22, 23]. However, in our study, Black men presented survival times similar to those of White men. This phenomenon might be explained by the fact that in the metastatic stage, there are few proactive treatments available to significantly change the oncological outcome of these patients. Interestingly, our study demonstrated that patients with an Asian or Pacific Islander background were associated with a better OS than White men, which is consistent with previous reports showing that Asian men have superior survival in de novo metastatic PCa than do men of other races [24]. Therefore, genomic diversities rather than treatments are most accountable for the different survival times between races [24]. It is known that allelic imbalance at 13q14 and 13q21 is significantly higher in Japanese patients than in Caucasians [25]. The frequency of the TMPRSS2-ERG fusion in Chinese patients is significantly lower than those patients from Western countries [26]. Furthermore, differences in diet (e.g., soy food consumption, which is popular in Asians and associated with a 25% to 30% reduced risk of PCa) and other lifestyle factors may also affect survival [24, 27].

### Lung was the most frequent site of synchronous metastases

Our study shows that in PCa patients with bone metastases, approximately 10% of patients have synchronous distant metastases at other sites. Among these sites, the lung was the most frequently involved extra-skeletal organ, followed by the liver, whereas brain metastasis was relatively uncommon. These results are in line with those of former studies [18, 28]. A previous autopsy study of 1589 PCa patients also demonstrated that lung was the second most frequent site of involvement, following bone [29]. However, there is very few published researches on the mechanisms of lung metastasis in prostate cancer. Several biological processes, such as hemodynamics, bone-specific signaling interactions, and the “seed and soil” hypothesis, were attributable to the bone metastasis of PCa [30]. Lung metastasis of breast cancer, another primary of high incidence and metastatic rate, is often explained by the “seed and soil” hypothesis [31]. To elucidate the mechanism of lung metastasis from PCa remains a challenge, which need further study to understand its pathogenesis and prognosis.

### Liver metastases were associated with poor OS

As described in the results section, we observed significant associations between the sites of metastases and patients’ survival. In particular, patients with liver metastases showed a poorer prognosis than those with other types of metastases. It is important to understand which of the two factors, the number of metastatic sites and the specific site of metastases, has a greater impact on the prognosis of patients. For this purpose, we analyzed PCa patients with lung-only, brain-only, and liver-only metastases using Kaplan–Meier survival curves and log-rank tests and found that patients with liver metastases showed the worst prognosis (Additional file 1: Fig. S1A). Kaplan–Meier survival curves and log-rank tests showed that as long as a patient had liver metastases, no matter how many other sites of metastases were present, the survival time was expected to be similarly short (Additional file 1: Fig. S1B). However, this phenomenon was not seen in patients with lung or brain metastases, as the number of metastatic sites significantly affected prognosis (Additional file 1: Fig. S1C and D).

It is known that PCa patients with liver metastases often have a lower Karnofsky Performance Status (KPS) scores [32]. Patients with low KPS scores cannot tolerate intensive systemic treatment. Another potential reason is that liver metastases from PCa tend to have a propensity for neuroendocrine differentiation, which is independently associated with poor survival outcomes [30, 33]. The neuroendocrine differentiation propensity in liver metastasis patients was also observed in our cohort. In PCa patients with bone-only metastases, neuroendocrine differentiation consisted of only 0.7%, whereas in bone and liver metastases, the rate rose up to 7% of the patients (Additional file 2: Table S1). These results suggest that we need to carefully evaluate the synchronous metastases of extra-skeletal organs prior to treatment in PCa patients with initial bone metastases.

### Chemotherapy improved OS in specific patient subpopulations

With regard to treatment, our study revealed that surgery, RT, and chemotherapy were uncommonly used in de novo PCa patients with bone metastases, with rates of only 9.6%, 22.6%, and 13.3%, respectively. This phenomenon was related to the fact that ADT had been the first line of treatment for metastatic PCa patients for many years [34].The addition of chemotherapy to impact PCa survival was first examined in castration-resistant prostate cancer (CRPC) in 2004. Since then, additional studies have described the role of chemotherapy increasingly earlier in disease presentation [35]. In recent years, several studies have supported the upfront use of chemotherapy at the initial diagnosis of metastatic prostate cancer. For example, in a large phase III trial, CHAARTED, the addition of 6-cycle docetaxel chemotherapy to ADT in men with newly diagnosed hormone-naive PCa resulted in a 14-month improvement in median survival (57.6 vs. 44 M) [36]. And the phase III study of ADT alone or with docetaxel in non-castrate metastatic prostate cancer (GETUG-AFU 15) found that, the addition of 9-cycle docetaxel chemotherapy to ADT in radiologically confirmed metastatic PCa resulted in modest OS benefits (60 vs. 54 M) [37]. Therefore, only in recent years has chemotherapy been recommended by the NCCN and EAU guidelines for certain metastatic PCa patients who are fit enough to receive the combination treatment at initial diagnosis [10, 11]. In our study, similarly, when all patients were pooled for multivariate Cox analysis, chemotherapy had a positive role in reducing the risk of mortality, with an HR of 0.848.

Interestingly, landmark analysis showed that, compared with bone-only metastases patients receiving no chemotherapy, patients receiving chemotherapy had a significantly longer OS before the landmark point of 35 months. However, significantly shorter OS after 35 months was otherwise observed. The reasons may include (1) the Kaplan–Meier analysis was not adjusted for age, marriage, race, PSA, Gleason score, etc., whereas the results of the multivariate Cox regression were adjusted for the above variable and showed that chemotherapy prolonged OS; and (2) as recommended by the NCCN and EAU guidelines, patients with a low-volume metastatic disease have less certain benefit from early-delivered chemotherapy. Therefore, we might assume that the majority of patients receiving chemotherapy tend to be of high-volume disease, which may affect patients’ long-term survival. In addition, it is noted that more than 93% of chemotherapy patients deceased before 35 months, while only less than 7% of chemotherapy patients stayed alive after 35-month. Therefore, the power of Kaplan–Meier analysis may be limited.

Another interesting result was that, although chemotherapy was an independent prognostic factor in patients with only bone, bone and lung, and bone and liver metastases, it was not in patients with bone and brain, or bone metastases and ≥ 2 other sites (including some brain metastases) of metastases. These results indicate that chemotherapy only improves OS in patients without brain metastases, which might be explained by the effect of the blood–brain barrier on the efficacy of chemotherapy [38]. Experimentally, van der Veldt et al. [39] once reported very low uptake of docetaxel in the brain of PCa patients receiving chemotherapy.

### RP and BT improved OS in patients with bone-only metastases

In our study, when all patients were pooled for multivariate Cox analysis, neither surgery nor RT had an effect in reducing the risk of mortality, with HRs of 1.006 and 0.955, respectively. Therefore, all patients were stratified according to the sites of metastases and treatment modalities for further evaluation of survival benefits. Although, in our study, in the group of PCa patients with bone-only metastases, only 206 and 94 patients underwent RP and BT, respectively. Interestingly, RP and BT both presented significant benefits in reducing the risk of mortality, with HRs of 0.339 and 0.567, respectively. However, similar to PCa patients with bone-only metastases, RP and BT were rarely used in PCa patients with bone metastases and other sites of metastases. Due to the small amount of data, multivariate Cox analysis was not possible. In 2014, Culp et al. [12] reported that local therapy with RP and BT appeared to confer a survival benefit for metastatic PCa. Our study, therefore, provided meaningful support to the study by Culp et al., as we pointed out that the benefits of RP and BT were mainly observed in PCa patients with bone-only metastases. Due to the small sample size, further investigation is warranted to validate these findings.

EBRT to the primary tumor was strongly discouraged for metastatic PCa patients in the NCCN and EAU guidelines in the versions issued prior to 2019. EBRT was often used to palliate metastatic bone pain, spinal cord compression, and obstructive symptoms due to the primary tumor [40, 41]. However, in the most recent version of the NCCN and EAU guidelines, EBRT combined with ADT was recommended for metastatic PCa patients who had a low metastatic burden according to the CHAARTED criteria (a high metastatic burden was defined as four or more bone metastases with one or more metastases outside of the vertebral body or pelvis, or visceral metastases, or both; all other assessable patients were considered to have a low metastatic burden) [10, 11]. This updated recommendation was based on the STAMPEDE phase 3 randomized trial, which was reported in 2018 and suggested that prostate RT improves OS for men with metastatic PCa who have a low metastatic burden but not for all patients [42]. Since the SEER database does not provide information on the sites of RT, we assumed that BT was RT for the primary lesion, while EBRT may have been RT for the primary or metastatic lesion. This finding may explain why the BT group showed a longer OS, while the EBRT group showed no OS advantage in de novo PCa patients with bone-only metastases in our study.

Similar to other studies that have utilized the SEER database as their data source, our study did have certain limitations. First, only four distant sites of metastases were assessed, and details on the metastases, such as the sizes and exact metastatic lesion quantity in specific organs, were lacking. Second, all information on metastases was from the initial diagnosis, and there was a lack of follow-up information. Third, although the SEER data included information regarding the use of surgery, RT, and chemotherapy, the details of these therapies (i.e., surgical margins, radiation dose, radiation to the primary tumor, chemotherapy regimen and chemotherapy sequence) were not recorded in the database. Fourth, some other treatment information that might be important for prognosis, such as ADT and abiraterone acetate, was lacking.

## Conclusions

In summary, our study found that age, race, tumor grade, PSA, Gleason score, sites of synchronous extra-skeletal metastases, as well as treatment modalities affected OS in newly diagnosed PCa patients with bone metastases. Specifically, (1) Asian or Pacific Islander ethnic background was associated with prolonged OS, which may be a combined result of genomic and lifestyle factors; (2) lung was the most frequent site of synchronous metastases; (3) liver metastases were associated with the worst prognosis; (4) chemotherapy improved OS in specific patient subpopulations without brain metastasis; and (5) RP and BT improved OS in patients with bone-only metastases, which need to be validated in further investigations due to limited cohort size of the current study.

## Supplementary information

**Additional file 1: Figure S1.** Kaplan-Meier survival curves and log-rank tests showing overall survival in PCa patients with only one site metastases, lung metastases, brain metastases, and liver metastases. A: Patients with liver metastases showed the worst prognosis. B: As long as a patient had liver metastases, no matter how many other sites of metastases were present, the survival time was expected to be similarly short. C: OS differences between patients with lung-only metastases (1 site), lung and another site metastases (2 sites), and lung and ≥ 2 other sites metastases (≥ 3 sites). D: OS differences between patients with brain-only metastases (1 site), brain and another site metastases (2 sites), and brain and ≥ 2 other sites metastases (≥ 3 sites).

**Additional file 2: Table S1.** Pathology types (ICD-0-3) of PCa patients with bone-only metastases, bone and lung metastases, bone and liver metastases, bone and brain metastases, and bone and ≥ 2 other sites metastases.

## Data Availability

The data that support the findings of this study are openly available in the Surveillance, Epidemiology, and End Results (SEER) database of the National Cancer Institute at https://seer.cancer.gov/.

## Abbreviations

ADT: androgen deprivation therapy
BT: brachytherapy
CHAARTED: Chemohormonal Therapy Versus Androgen Ablation Randomized Trial for Extensive Disease in Prostate Cancer
EAU: European Association of Urology
EBRT: external-beam radiation
NCCN: National Comprehensive Cancer Network
OS: overall survival
PCa: prostate cancer
PSA: prostate-specific antigen
RP: radical prostatectomy
RT: radiotherapy
SEER: Surveillance, Epidemiology, and End Results
