# Supplementary figures and images for: Sites of synchronous distant metastases and prognosis in prostate cancer patients with bone metastases at initial diagnosis: a population-based study of 16,643 patients
Source: Clin Transl Med. 2019 Nov 29;8:30. doi: 10.1186/s40169-019-0247-4 (PMC6884608; doi:10.1186/s40169-019-0247-4)

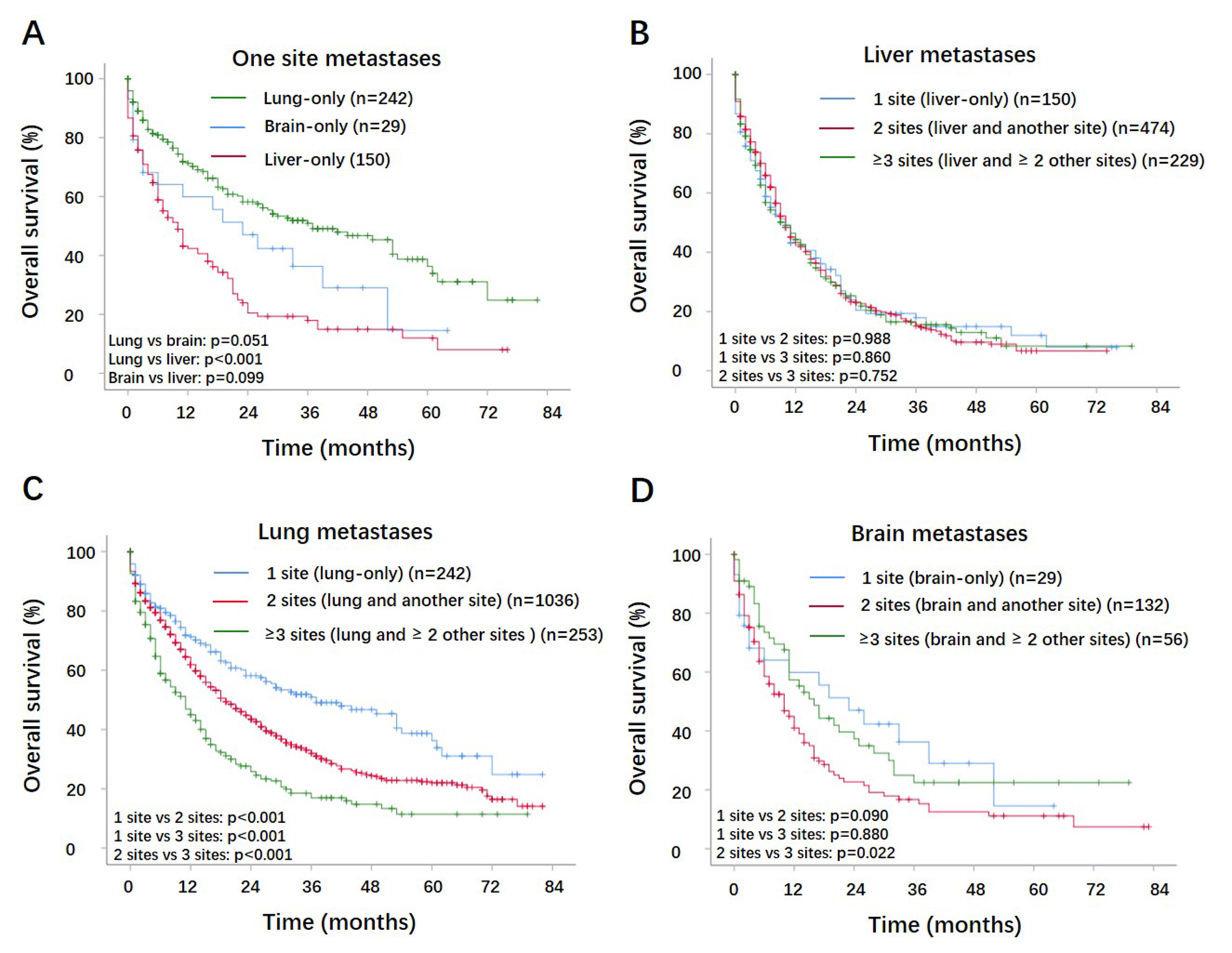

Supplement: Supplementary file 1 — Additional file 1: Figure S1. Kaplan-Meier survival curves and log-rank tests showing overall survival in PCa patients with only one site metastases, lung metastases, brain metastases, and liver metastases. A: Patients with liver metastases showed the worst prognosis. B: As long as a patient had liver metastases, no matter how many other sites of metastases were present, the survival time was expected to be similarly short. C: OS differences between patients with lung-only metastases (1 site), lung and another site metastases (2 sites), and lung and ≥ 2 other sites metastases (≥ 3 sites). D: OS differences between patients with brain-only metastases (1 site), brain and another site metastases (2 sites), and brain and ≥ 2 other sites metastases (≥ 3 sites). [file 40169_2019_247_MOESM1_ESM.jpg]
