# Supplementary material for: Sites of synchronous distant metastases and prognosis in prostate cancer patients with bone metastases at initial diagnosis: a population-based study of 16,643 patients
Source: Clin Transl Med. 2019 Nov 29;8:30. doi: 10.1186/s40169-019-0247-4 (PMC6884608; doi:10.1186/s40169-019-0247-4)
Supplement: Supplementary file 2 — Additional file 2: Table S1. Pathology types (ICD-0-3) of PCa patients with bone-only metastases, bone and lung metastases, bone and liver metastases, bone and brain metastases, and bone and ≥ 2 other sites metastases. [file 40169_2019_247_MOESM2_ESM.docx]

**Table S1.** Pathology types (ICD-0-3) of PCa patients with bone-only metastases, bone and lung metastases, bone and liver metastases, bone and brain metastases, and bone and ≥ 2 other sites metastases.

|  | **Pathology type (ICD-0-3)** | **Bone-only**  **(n=14872)** | **Bone and lung**  **(n=976)** | **Bone and liver**  **(n=414)** | **Bone and brain**  **(n=126)** | **Bone and ≥2 other sites**  **(n=255)** |
| --- | --- | --- | --- | --- | --- | --- |
| **1. Neuroendocrine differentiation related tumor** | | **103 (0.7%)** | **18 (1.8%)** | **29 (7.0%)** | **4 (3.2%)** | **19 (7.5%)** |
|  | 8013/3 Large cell neuroendocrine carcinoma | 0 (0.0%) | 0 (0.0%) | 0 (0.0%) | 0 (0.0%) | 1 (0.4%) |
|  | 8041/3 Small cell carcinoma, NOS | 42 (0.3%) | 8 (0.8%) | 17 (4.1%) | 2 (1.6%) | 10 (3.9%) |
|  | 8045/3 Combined small cell carcinoma | 11 (0.1%) | 1 (0.1%) | 3 (0.7%) | 0 (0.0%) | 1 (0.4%) |
|  | 8244/3 Mixed adenoneuroendocrine carcinoma | 0 (0.0%) | 0 (0.0%) | 0 (0.0%) | 0 (0.0%) | 1 (0.4%) |
|  | 8246/3 Neuroendocrine carcinoma, NOS | 22 (0.1%) | 5 (0.5%) | 4 (1.0%) | 2 (1.6%) | 4 (1.6%) |
|  | 8574/3 Adenocarcinoma with neuroendocrine differentiation | 28 (0.2%) | 4 (0.4%) | 5 (1.2%) | 0 (0.0%) | 2 (0.8%) |
| **2. 8140/3 Adenocarcinoma, NOS** | | 12392 (83.3%) | 802 (82.2%) | 309 (74.6%) | 97 (77.0%) | 187 (73.3%) |
| **3. Others** | | 2377 (16.0%) | 156 (16.0%) | 76 (18.4%) | 25 (19.8%) | 48 (18.8%) |

**Note:** The three pathology types were compared by the chi-square test (p<0.001)**.**
